# Inhibition of porcupine prolongs metastasis free survival in a mouse xenograft model of Ewing sarcoma

**DOI:** 10.18632/oncotarget.19432

**Published:** 2017-07-21

**Authors:** Masanori Hayashi, Alissa Baker, Seth D. Goldstein, Catherine M. Albert, Kyle W. Jackson, Gregory McCarty, Ulf D. Kahlert, David M. Loeb

**Affiliations:** ^1^ Sidney Kimmel Comprehensive Cancer Center, Johns Hopkins University, Baltimore, MD, USA; ^2^ Neurosurgical Clinic, University Medical Center Düsseldorf, Düsseldorf, Germany; ^3^ Seattle Children’s Hospital, University of Washington, Seattle, WA, USA

**Keywords:** metastasis, patient-derived xenograft, preclinical model, Wnt signaling, Ewing sarcoma

## Abstract

The most pressing unmet clinical need for patients with Ewing sarcoma (ES) is the prevention and treatment of metastasis. The Wnt signaling pathway regulates a number of cellular functions associated with metastasis, including proliferation, motility, and stem cell self-renewal. Functional interaction between Wnt ligands and their receptors requires palmitoylation by Porcupine (Porcn), making this an ideal therapeutic target. We studied the effect of WNT974, a potent, selective Porcn inhibitor, on ES metastasis. *In vitro*, WNT974 does not affect ES proliferation or sarcosphere formation, but suppresses multiple transcriptional regulators of metastasis and inhibits cell migration. *In vivo*, in an orthotopic implantation/amputation model of spontaneous distant metastasis, single agent WNT974 treatment leads to a significant delay in formation of lung metastasis and a substantial improvement in post-amputation survival without a major effect on primary tumor growth. The drug produces no survival benefit in a tail vein injection model, supporting the hypothesis that WNT974 inhibits early steps in the metastatic cascade, such as migration and invasion. Our findings strongly implicate Wnt signaling in the early steps of ES metastasis and demonstrate that WNT974 has the potential to significantly improve the survival of ES patients through the specific inhibition of metastasis.

## INTRODUCTION

With the introduction and intensification of conventional chemotherapy, long term survival for patients with localized Ewing sarcoma (ES) has dramatically improved, from the 8-20% seen in the pre-chemotherapy era to close to 70% in the modern era [1-3]. Although this is one of the exemplary success stories of chemotherapy in solid tumors, as many as 30% of patients with localized disease will not be cured, despite multimodal treatment, due to the development of metastases. Similarly, although the majority of ES patients presenting with metastatic disease achieve a complete remission, cure rates still hover at about 20%, and there has been no improvement in the past 3 decades [4]. These data reveal the glaring weakness of currently available therapeutics - our current treatments are highly effective for the primary tumor, but they do not specifically target the complicated metastatic cascade.

The Wnt signaling pathway is a key regulator of adult stem cell function, especially self-renewal and proliferation, as well as being involved in the pathogenesis of several malignancies [5-8]. This pathway is extremely complicated, involving 19 Wnt ligands and 10 Frizzled (Fzd) receptors which transduce signals *via* both canonical and non-canonical pathways [8]. While most oncologic research has focused on the role of β-catenin (canonical pathway), the non-canonical pathways have attracted attention more recently because of a role in cell motility and possibly in metastasis. The complexity of the Wnt signaling pathway has been a substantial obstacle to therapeutic targeting. Recognition that secretion of Wnt ligands and functional interaction with their receptors requires ligand palmitoylation by the acyl transferase, Porcupine (Porcn), led to the development of WNT974, a potent, selective, orally bioavailable small molecule Porcn inhibitor [9].

The importance of Wnt signaling in ES biology has been controversial, as ES does not demonstrate β-catenin-dependent proliferation [10-12]. Several groups have reported the expression of different Wnt ligands and Fzd receptors in ES cell lines, and have demonstrated that morphologic changes or enhanced motility and invasion can be induced by exogenous Wnt ligands [13-15]. WNT974 presents a unique opportunity to determine the role of Wnt signaling in ES biology by targeting both the canonical and non-canonical pathways activated by endogenous ligands. We used WNT974 to inhibit Wnt signaling in ES, both *in vitro* (cell lines) and *in vivo* (patient-derived xenografts). We observed that WNT974 suppressed expression of a significant number of genes previously implicated in motility, invasion, and metastasis, resulting in phenotypic changes characterized by decreased motility and invasion *in vitro*. In a mouse xenograft model, there was no significant effect on primary tumor growth, but we observed a striking delay in the development of hematogenous metastases which translated into prolonged survival. Our results suggest that WNT974 may represent an important treatment advance by specifically targeting ES metastasis.

## RESULTS

### WNT974 downregulates Wnt target genes in ES cell lines

**Figure 1:**
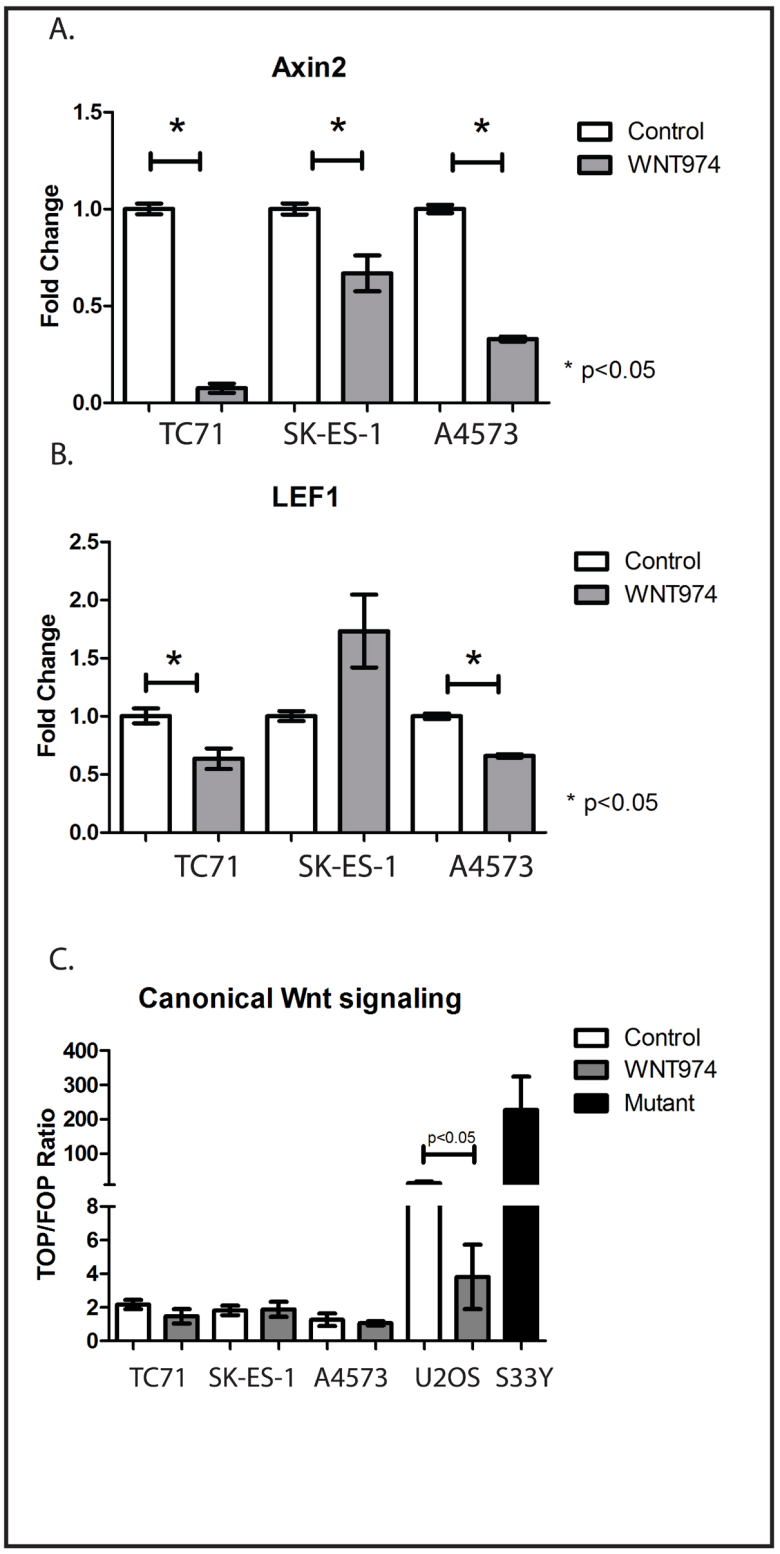
WNT974 inhibits Wnt signaling in ES cell lines The indicated cell lines were treated with or without 1 μM WNT974 for 48 hours prior to mRNA isolation. Quantitative RT-PCR was performed using primers specific for Axin2 **A.** and LEF1 **B.** Expression was normalized to beta-2 microglobulin by the ΔΔCt method. Data shown are the mean relative expression of triplicate samples. Error bars represent standard error of the mean. Experiments were repeated 3 times, and representative results are presented. To evaluate the level of β-catenin-dependent signaling, the same cell lines, as well as U2OS osteosarcoma cells, were transfected with the TOP/FOP Flash plasmids. SK-ES-1 cells stably transduced to constitutively express active mutant β-catenin (S33Y) are included as a positive control. Luciferase assays were performed as described, and the ratio of TOP luciferase to FOP luciferase normalized by Renilla luciferase activity is presented. Error bars represent standard error of the mean. Experiments were repeated 3 times, and representative results are presented.

WNT974 has been shown to inhibit the expression of Wnt pathway target genes in breast cancer cell lines, but we initially validated the ability of WNT974 to inhibit Wnt signaling in ES cells as well. Expression of Axin 2 and LEF1 are generally considered to be evidence of active Wnt signaling [9]. We treated TC71, SK-ES-1, and A4573 cells with either 1µM WNT974 or vehicle control for 48 hours, and expression of Axin 2 and LEF1 were quantified by RT-PCR. This dosing regimen was based on published pharmacokinetic data [9]. As anticipated, expression of both genes was inhibited by WNT974 (Figure 1A, 1B) consistent with the ability of this drug to inhibit Wnt signaling.

Functional interaction between Wnt ligands and their receptors can activate a variety of intracellular signaling pathways, some of which, termed “canonical,” culminate in β-catenin-mediated transcriptional changes, and others of which, termed “non-canonical,” are independent of β-catenin. To determine the relative contribution of β-catenin-dependent pathways to Wnt signaling in ES cells, we utilized the TOP/FOP Flash reporter assay. TC71, SK-ES-1, and A4573 cells were transfected with a plasmid containing a luciferase cDNA under the transcriptional control of a promoter with tandem repeats of the TCF/LEF response element (TOP) or mutant versions of the element (FOP). The ratio of TOP-luciferase to FOP-luciferase reflects the relative activity of the β-catenin-dependent pathway. A Renilla luciferase reporter was cotransfected for normalization. As controls, we included the osteosarcoma cell line U2OS, which has previously been demonstrated to have strong, endogenous β-catenin-dependent signaling [16], and SK-ES-1 cells stably transduced to express mutant (S33Y), constitutively active β-catenin. The SK-ES-1-S33Y cells and U2OS cells had very high ratios of TOP to FOP luciferase signals, and WNT974 decreased this activity in U2OS cells by 4-fold (15.2±4.2 to 3.8±1.9; Figure 1C). All 3 ES cell lines had ratios indicating less activity than in the WNT974-treated U2OS cells, and WNT974 had no effect on these cell lines (Figure 1C). Taken together, these data suggest that, although WNT974 inhibits Wnt signaling in ES cell lines, the “canonical,” β-catenin-dependent pathway plays at most a minor role in these cell lines.

### WNT974 inhibits ES migration but not proliferation or sphere formation

**Figure 2:**
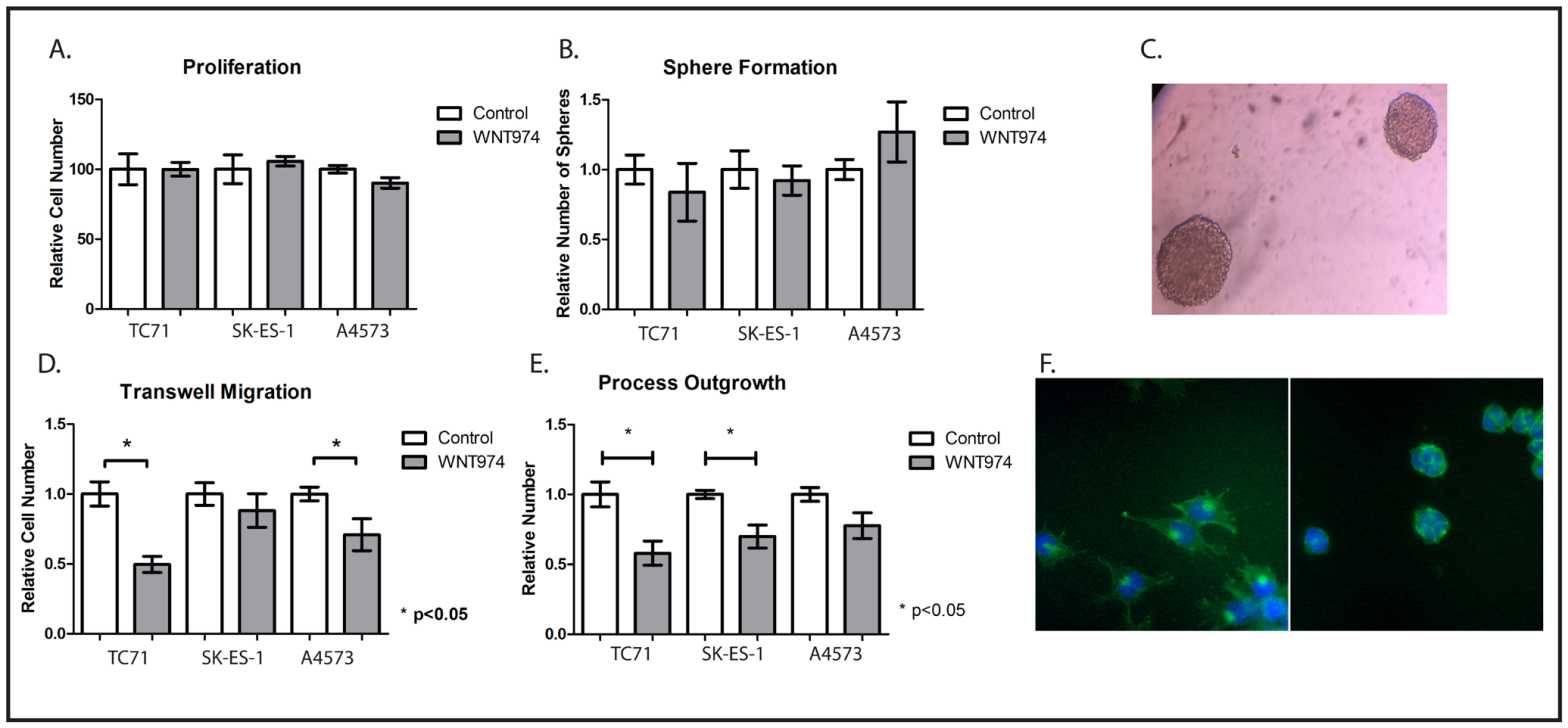
*In vitro* phenotypic effects of WNT974 on ES cell lines **A.** The indicated cells were treated with or without 1 μM WNT974 for 48 hours, and relative cell number was quantified using the Cell Counting Kit-8. Data shown are the mean relative cell number, with untreated cells defined as 100%, from experiments performed in triplicate. Error bars represent standard error of the mean. Experiments were repeated 3 times and representative results are presented. **B.** The indicated cells were grown under nonadherent conditions for 7 days in the presence or absence of 1 μM WNT974 and spheres were counted manually. Data shown are the mean relative number of spheres per well, from triplicate wells, with untreated cells defined as 1.0. Error bars represent standard error of the mean. Experiments were repeated 3 times and representative results are presented. **C.** Representative TC71 sarcospheres are shown (40x magnification). **D.** Migration of the indicated cell lines in a Boyden chamber assay was quantified in the presence or absence of 1 μM WNT974. Data shown are the relative number of cells migrating through the barrier, performed in triplicate, with untreated cells defined as 1.0. Error bars represent standard error of the mean. Experiments were repeated 3 times and representative results are presented. **E.** The indicated cells were grown for 48 hours in the presence or absence of 1 μM WNT974 and then fixed and stained with the Cytopainter F-Actin Staining Kit. Long cytoplasmic extensions were counted and data shown are the relative number of extensions per cell, quantified from triplicate wells and normalized to a value of 1.0 for control cells. Error bars represent standard error of the mean. Experiments were repeated 3 times and representative results are presented. **F.** Representative images of SK-ES-1 cells stained with the Cytopainter F-Actin Staining Kit after growing in vehicle (left image) or 1 μM WNT974 (right image) for 48 hours (100x magnification).

In other tumor types, Wnt signaling has been implicated in proliferation, stem cell function, and motility, but the role of endogenous Wnt signaling in ES remains unclear. We next used WNT974 to determine which of these functions is regulated by Wnt signaling in ES cells in culture. We treated TC71, SK-ES-1, and A4573 cells with either 1µM WNT974 or vehicle control and found no effect on cell number at 48 hours (Figure 2A). We next assessed sphere formation under nonadherent conditions after one week of incubation, an assay that has been used as an *in vitro* surrogate for stem cell function. Again, 1 μM WNT974 had no effect on sphere forming activity of TC71, SK-ES-1, or A4573 cells (Figures 2B and 2C). Because exogenous Wnt ligand stimulation has been implicated in morphologic changes and in migration [15, 17] through regulation of actin cytoskeletal dynamics, we investigated whether WNT974 affected the migratory behavior of ES cells. Interestingly, we found that WNT974 significantly decreased migration of TC71 and A4573 cells in a Boyden chamber assay (Figure 2D) and, reflecting a direct effect on the actin cytoskeleton, decreased process formation in TC71 and SK-ES-1 cells (Figures 2E, 2F) as determined by phalloidin staining.

### WNT974 downregulates metastasis-related genes in **Ewing** sarcoma cell lines

**Figure 3:**
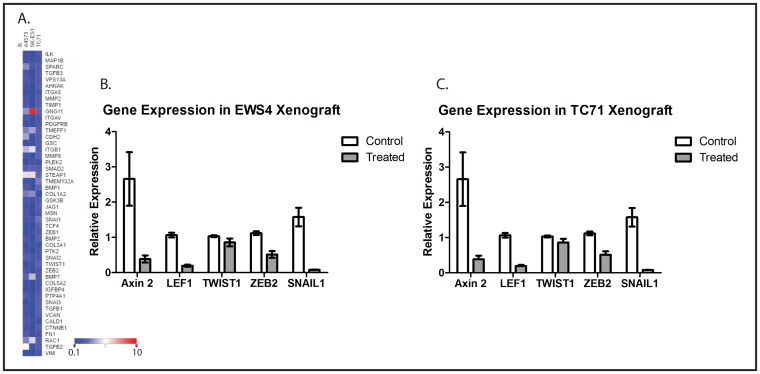
WNT974 downregulates Wnt target genes and metastasis-related genes in ES cell lines and xenografts **A.** The indicated cell lines were treated with or without 1 μM WNT974 for 48 hours, and mRNA was isolated, reverse transcribed, and analyzed using an EMT gene-focused PCR array. Expression of the indicated genes in treated cells was quantified relative to control cells by the ΔΔCt method after normalization based on beta-2 microglobulin expression. A heatmap of the results is presented. The deepest blue represents expression level of 0.1 in treated cells compared with control, and the deepest red represents an expression level of 10.0 in treated cells compared with control. Mice bearing the EWS4 PDX **B.** or a xenograft derived from the TC71 cell line **C.** were treated with or without WNT974 at 5mg/kg/dose twice daily for 3 days. Tumors were harvested and expression of the indicated genes evaluated by RT-PCR, using beta-2-microglobulin expression for normalization. Fold expression refers to expression relative to tumors harvested from untreated mice. Data shown are the results of triplicate assays, and the error bar shows standard error of the mean. With the exception of TWIST1, each difference is statistically significant, with *p* < 0.01.

The epithelial-mesenchymal transition (EMT) is an essential step for otherwise adhesive cancer cells to go through the early steps of metastasis, including migration and invasion [18], and Wnt signaling has been implicated in regulating EMT in cancer [19]. As a mesenchymal tumor, it is not clear whether EMT plays a role in ES metastasis at all, but many genes implicated in EMT, including TWIST1, ZEB2, and SNAIL1, have also been implicated in ES metastasis [17, 20, 21]. To determine whether WNT974 affects the expression of metastasis-related genes in ES cells, we used a commercially available PCR array which simultaneously evaluates expression of genes that are both up- and downregulated upon EMT, as well as genes that drive this process. This array was chosen because of the significant overlap between genes implicated in metastasis and genes implicated in EMT, including TWIST1, ZEB2, and SNAIL1. Focusing on the genes represented on the array that are positively associated with metastasis, we found that 1 μM WNT974 significantly downregulates these genes across multiple ES cell lines (Figure 3A). These results, in the context of our other *in vitro* data, suggest that Wnt signaling affects ES migration (but not proliferation) *via* a global effect on expression of metastasis-associated genes, findings that support the hypothesis that preferentially affect ES metastasis, rather than primary tumor growth, *in vivo*.

To confirm that WNT974 inhibits expression of downstream targets of Wnt signaling and of metastasis-associated genes *in vivo* as well as *in vitro*, we implanted fragments of a Ewing sarcoma PDX, designated EWS4, as well as fragments of a xenograft derived from TC71 cells, into the pretibial space of NOD/SCID/IL-2Rγ-null (NSG) mice. After allowing time for initial tumor growth, mice were treated with WNT974 at 5mg/kg/dose twice daily for 3 days. Tumors were harvested, and expression of a small panel of genes was evaluated by quantitative RT-PCR. Consistent with our *in vitro* results, WNT974 significantly suppressed expression ofAxin2 and LEF1 (confirming inhibition of Wnt signaling) and ZEB2 and SNAIL1 (key regulators of metastasis), with a modest effect on TWIST1 (Figures 3B and 3C).

### WNT974 inhibits metastasis *in vivo*

Because Wnt signaling has been implicated in stem cell function and tumor initiating activity, we investigated the effect of WNT974 on ES tumor initiation. One thousand TC71 cells, pretreated *in vitro* with or without 1µM WNT974, were implanted in Matrigel subcutaneously into NSG mice that were also pretreated with or without WNT974 at 5mg/kg/dose twice daily for 3 days. All 5 mice in both cohorts developed tumors at the same time, showing no difference in tumor initiating activity. This, along with our data on *in vitro* sphere formation (Figure 2B) argues against a direct effect of WNT974 on Ewing sarcoma stem cell/tumor initiating cell function.

**Figure 4:**
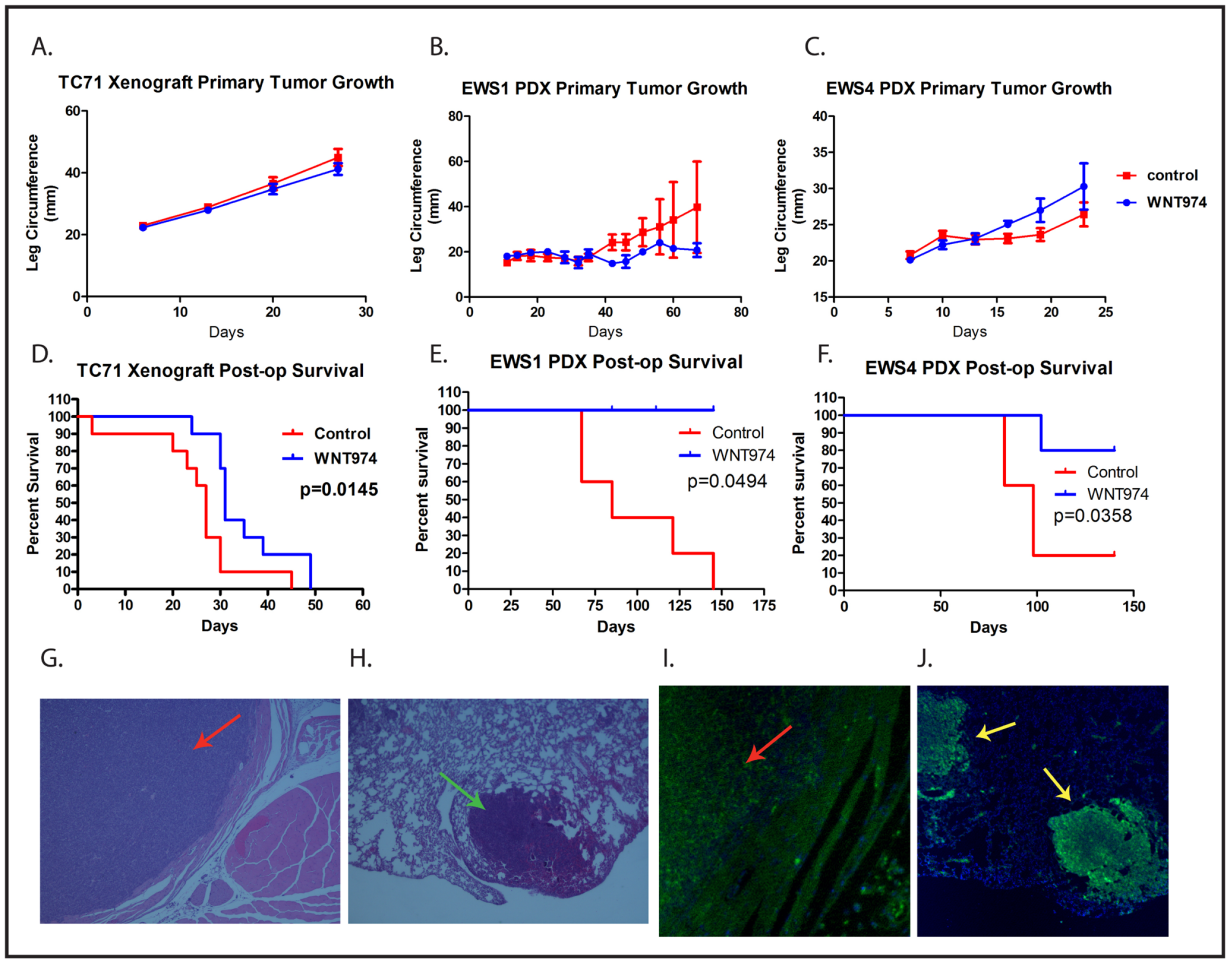
WNT974 prolongs survival of PDX-bearing mice without affecting primary tumor growth Mice were implanted with fragments of a TC71 xenograft **A.** and **D.**, the EWS1 PDX **B.** and **E.** or the EWS4 PDX **C.** and **F.** and then treated with or without WNT974 at 5mg/kg/dose twice daily. Tumor growth was quantified twice weekly **A.**-**C.** After amputation, mice were followed until death **D.**-**F.** Tumor growth data show the mean leg circumference, with error bars showing standard deviation. Differences were evaluated for statistical significance by 2-way ANOVA but none of the growth curves showed a statistically significant difference. Kaplan-Meier survival curves were tested for significance by the log-rank test. Histological and immunofluorescent evaluation of the primary tumor **G.** and **I.** and of metastases isolated from the lung at necropsy **H.** and **J.** demonstrate a small round blue cell tumor **G.** and **H.** of human origin (based on immunofluorescent staining with anti-human mitochondrion antibodies; **I.** and **J.**, consistent with Ewing sarcoma. Panels **G.** and **H.** show a primary tumor (red arrow) and a pulmonary metastasis (green arrow) stained with haematoxylin and eosin at 4x **G.** and 20x **H.** magnification. Panels **I.** and **J.** show a primary tumor (red arrow) and 2 pulmonary metastases (yellow arrows) stained with Alexa Fluor 488 -labeled anti-human mitochondrion antibody (green) and counterstained with DAPI (blue) at 4x **I.** and 20x **J.** magnification.

To evaluate the effect of WNT974 on ES primary tumor growth and metastasis *in vivo*, tumor fragments were implanted into the pretibial space of NSG mice as previously described [22]. After ascertaining initiation of tumor growth, mice were treated with or without WNT974 at 5 mg/kg/dose twice daily by gavage. Although we saw a modest, and not statistically significant, effect on the growth of the EWS1 PDX, we saw no change in growth of the EWS4 PDX, nor on growth of a TC71 cell line xenograft (Figures 4A-4C). There was no perioperative mortality associated with hindlimb amputation. Despite the lack of an effect on primary tumor growth, mice treated with WNT974 had a substantial prolongation of survival after amputation of the primary tumor (Figures 4D-4F). Examination of metastatic lesions at necropsy confirmed that mice died due to metastatic Ewing sarcoma (Figures 4G - 4J). Thus, WNT974 prolongs disease-specific survival of mice bearing ES xenografts due to an effect not on primary tumor growth, but on the development of metastatic disease.

**Figure 5:**
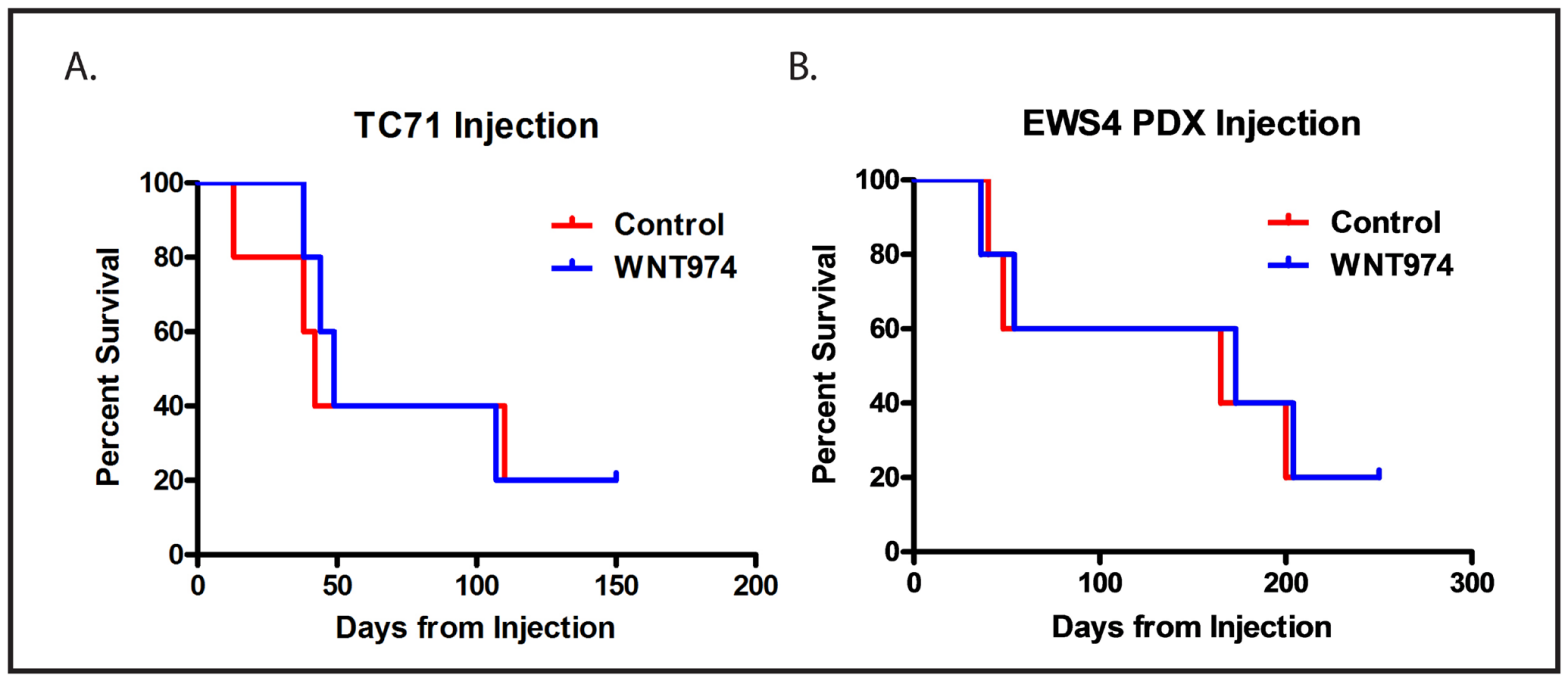
WNT974 does not prolong survival in a tail vein injection model Ten thousand TC71 cells **A.** or cells from a single cell suspension made from the EWS4 PDX **B.** were injected into the tail vein of mice treated with or without (control) WNT974 at 5mg/kg/dose twice daily. Mice were followed until death from metastatic disease.

The prolongation of survival in the absence of an effect on tumor initiation or growth suggests that WNT974 inhibits metastasis by affecting a process other than proliferation or stem cell function. Distant hematogenous metastasis is a complex, multi-step process. To determine whether WNT974 affects an early step in the process (prior to intravasation into the vasculature to form circulating tumor cells) or a late step in the process (extravasation and establishment of metastases in a distant site), we investigated whether the drug has an effect on development of metastases using a tail vein injection model. In this model, cells are injected directly into the circulation, bypassing the earliest steps in the metastatic cascade, thus modeling the late steps of metastasis. Ten thousand cells, either TC71 or dissociated EWS4, were treated *in vitro* with or without 1µM WNT974 for 48 hours and then were injected into the tail vein of NSG mice either pretreated 5mg/kg/dose twice daily for 3 days with WNT974 or not. The WNT974 cohort continued to receive drug throughout the experiment. In contrast to our orthotopic implantation/amputation model, WNT974 had no effect on establishment of metastases nor on survival of mice injected with cells *via* the tail vein (Figure 5). Thus, WNT974 is affecting an early, rather than a late, step in the metastatic cascade.

**Figure 6:**
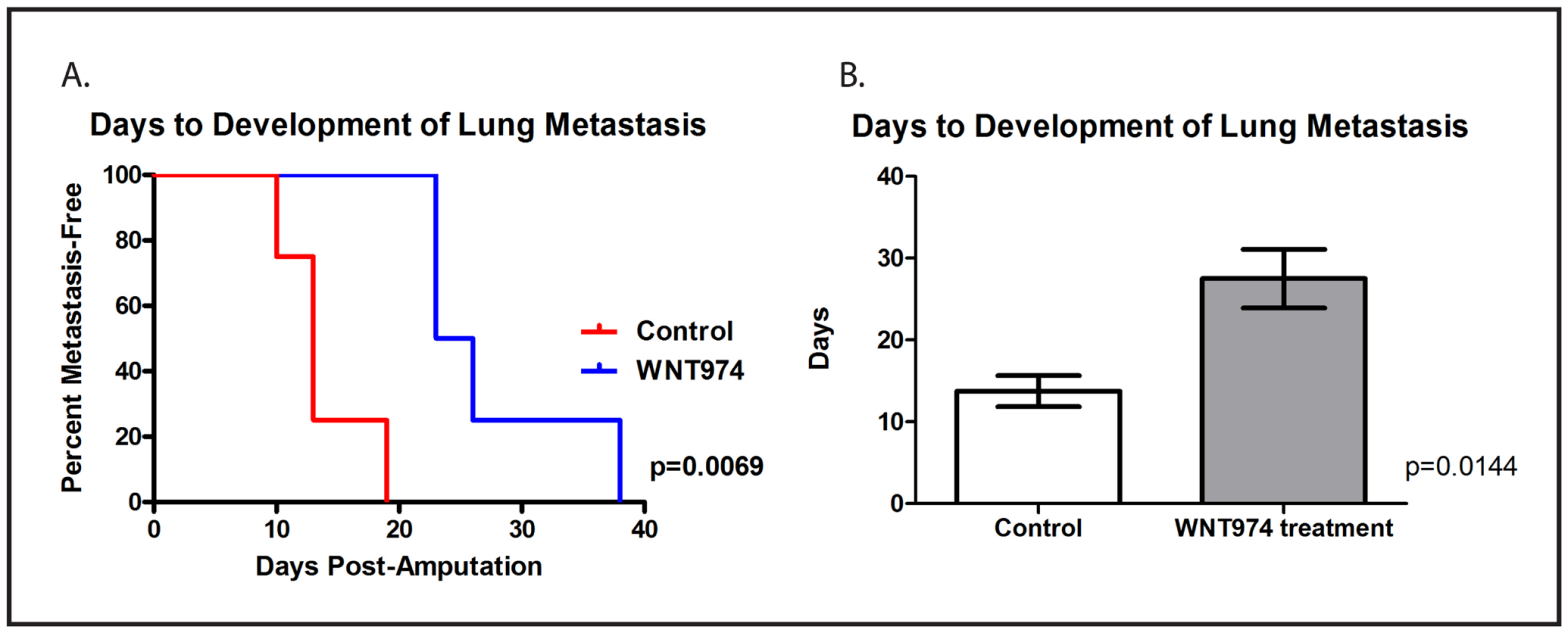
WNT974 prolongs the time to development of lung metastasis Fragments of a TC71 xenograft were implanted in the pretibial space of NSG mice which were then treated with or without WNT974 at 5mg/kg/dose twice daily. Mice were imaged weekly MRI using the Bruker 9.4T horizontal bore spectrometer, and images were evaluated by an investigator blinded to the status of the mice for the presence or absence of lung metastases. Differences in time to the development of first lung metastasis were evaluated by log-rank test **A.** or Student’s t test **B.**, and both methods confirmed a statistically significant difference.

Finally, although WNT974 prolongs the survival of mice implanted with ES fragments in the pretibial space, many of the treated mice did eventually succumb to distant metastatic disease, most commonly in the lung. Metastasis to kidneys and the retroperitoneum were also observed. To assess whether WNT974 delays the development of lung metastasis formation or simply slows the rate of growth of these metastases once formed (either of which would be expected to translate into a survival advantage), we repeated the orthotopic implantation/amputation experiment using the TC71 cell line xenograft with weekly lung MRIs performed following amputation. The MRI images were read by an investigator who was blinded to the treatment status, and the first day a lung metastasis was observed on MRI was determined for each animal. In this experiment, WNT974 significantly delayed the time to development of first lung metastasis (Figure 6). Taken together, these data suggest that WNT974 inhibits an early step in the metastatic cascade, which results in a delay in the development of metastases, which translates into prolonged survival.

## DISCUSSION

Although intensification of conventional therapy has produced significant improvement in the control of localized Ewing sarcoma, outcome for patients with metastatic disease remains dismal, with essentially no improvement for the past 4 decades [23, 24]. In this study, we describe the effects of WNT974, a Wnt signaling inhibitor that functions by inhibiting Porcn, the enzyme responsible for Wnt ligand palmitoylation, on the progression of Ewing sarcoma in an animal model of spontaneous distant metastasis. Although WNT974 did not inhibit tumor cell proliferation *in vitro*, nor localized tumor growth *in vivo*, inhibition of the expression of metastasis-associated genes and a significant effect on cell migration was seen *in vitro*, along with a significant delay in distant metastasis formation *in vivo*, which resulted in prolonged survival. The effect of WNT974 appears to be specifically directed against metastasis, supported both by *in vivo* and *in vitro* data, showing that this drug may represent a novel treatment strategy to inhibit Ewing sarcoma metastasis.

Metastasis is a complex multi-step process involving cell migration from the primary tumor, intravasation into the circulation, survival of circulating tumor cells (CTC), extravasation from the circulation, invasion into distant tissue, and establishment of a metastatic focus [25]. While it is clear that metastasis is the most critical aspect of Ewing sarcoma in determining a patient’s outcome, there is limited knowledge about the biology of Ewing sarcoma metastasis [26]. EMT is one of the key phenotypic transitions that allows carcinoma cells to migrate away from the primary tumor, invade through the surrounding tissue and into the vasculature to form a CTC. Since Ewing sarcoma is a mesenchymal tumor, EMT and its reversal, MET, have historically not been considered in this disease; however, emerging data support the importance of an “EMT-like” change that Ewing sarcoma cells undergo to develop distant metastasis [20, 27-29]. Regardless of the relevance of EMT to ES biology, it is clear that multiple EMT-related genes are also regulators of metastasis, and have been previously implicated in ES metastasis. Our current study provides new insight into targeting these changes in Ewing sarcoma cells through inhibition of Wnt signaling. While Wnt signaling is a very complex pathway, components of the pathway are key regulators of EMT and metastasis in several tumor types [30-32], and our study demonstrates that inhibition of Wnt signaling with WNT974 suppresses expression of genes associated with metastasis, many of which are considered drivers of EMT in carcinoma cells, providing a mechanistic explanation for our observation that WNT974 inhibits migration *in vitro* and metastasis *in vivo*.

Functional interaction between Wnt ligands and Wnt receptors triggers both β-catenin-dependent and β-catenin-independent intracellular signaling pathways. The relative contributions of these pathways to Wnt signaling in Ewing sarcoma remain unclear. Most published studies have involved exogenous Wnt ligands added to cultured Ewing sarcoma cells to determine a phenotypic effect, and these studies have focused on ligands that predominantly activate β-catenin-dependent signaling [13, 17]. Most studies evaluating endogenous signaling have similarly focused on β-catenin-dependent signaling because of the ease of demonstrating activation of this pathway *via* either nuclear staining of β-catenin by immunohistochemistry [17] or uncomplexed β-catenin by western blotting [12], but a survey of Wnt and Fzd expression in multiple cell lines demonstrated expression of ligands and receptors associated with both pathways [15]. WNT974 inhibits both β-catenin-dependent and β-catenin-independent signaling pathways and so demonstrates the importance of Wnt signaling in Ewing sarcoma metastasis regardless of the involvement of β-catenin. There are no available assays for generalized activation of β-catenin-independent signaling, since each pathway is distinct, but the TOP Flash assay is a standard method for demonstrating activity of the β-catenin-dependent pathway, and our results suggest that this plays only a minor role in Ewing sarcoma. Future work will focus on clarifying which β-catenin-independent pathways, including identifying key Wnt ligands and Fzd receptors, ultimately regulate Ewing sarcoma metastasis.

The best way to incorporate a metastasis-directed therapy such as WNT974 into the current treatment paradigms for Ewing sarcoma is not immediately obvious, but because this agent is already in clinical trials for a number of other malignancies, the pathway to Ewing sarcoma-specific clinical trials is open. Several potential translational approaches exist. WNT974 could be added to upfront therapy for patients with localized disease in an attempt to decrease the 30% rate of metastatic recurrence these patients currently suffer with chemotherapy alone. Alternatively, WNT974 could be tested as maintenance therapy, either for patients who present with metastases but achieve a complete response to initial chemotherapy (a population with an up to 85% chance of developing a metastatic recurrence) or for patients with initially localized disease, who have an almost 30% rate of metastatic relapse. The relatively favorable toxicity profile of this agent in the first reported Phase I study [33], as well as its specificity in targeting metastasis, rather than primary tumor growth, suggests that this is a potentially powerful new addition to the treatment of Ewing sarcoma patients.

## MATERIALS AND METHODS

### Cell lines and xenografts

ES cell lines TC71, A4573, SK-ES-1 were kind gifts from Jeffrey Toretsky (Georgetown University, Washington, DC). Cell line identity was confirmed by STR testing in the Johns Hopkins Genetic Resources Core Facility. Cells were cultured at 50-70% confluence in RPMI-1640 medium supplemented with 10% fetal bovine serum (FBS; Invitogen, Grand Island, NY) and were routinely confirmed to be *Mycoplasma* negative using the MycoAlert Plus *Mycoplasma* detection kit (Lonza, Allendale, NJ). Transplantable xenografts of primary human ES, EWS1 and EWS4, were a kind gift from Dr. Chand Khanna (National Institutes of Health, Bethesda, MD). The creation of these xenografts was approved by the Institutional Review Board of the National Institutes of Health, and patients gave informed consent for their creation.

### Animal experiments

NOD/SCID/IL-2Rγ-null (NSG) mice bred by Johns Hopkins University Research Animal Resources were implanted with 3 mm fragments of either a TC71 cell line xenograft or of the patient derived xenograft (PDX) EWS1 or EWS4 in the pre-tibial space as previously described [22]. Experiments were done with cohorts of either 5 or 10 mice per arm, and mice were randomly assigned to treatment or control arms. The circumference of the affected limb was measured every 3-4 days. When the leg circumference reached 15 mm, mice had their affected limbs amputated as previously described [22]. Mice implanted with the TC71 cell line xenograft or the EWS4 PDX were imaged by MRI using the Bruker 9.4T horizontal bore spectrometer with the assistance of the Johns Hopkins Small Animal Imaging Resource Program. Tail vein injections were performed by injecting 10,000 cells suspended in PBS directly into the tail vein. WNT974 treatment was administered 5mg/kg/dose twice daily by gavage, 10 times/week. Animals were euthanized when they exhibited signs and symptoms of pain and suffering, such as hunched posture, reluctance to move, weight loss > 20%, or a “body condition score” of 2/5 or worse. All experiments were approved by the Johns Hopkins University Institutional Animal Care and Use Committee.

### Proliferation assays

TC71, A4573, and SK-ES-1 cells were plated in 96 well plates in RPMI-1640 media with 10% FBS at a density of 2x10^4^ cells per well, and were treated with 1µM WNT974 or DMSO 1:1000 as a control. After 48 hours of incubation, cells were assessed for viability with the Cell Counting Kit-8 (Dojindo Molecular Technologies, Rockville, MD) per manufacturer’s instructions.

### Migration assays

TC71, A4573, and SK-ES-1 cells were plated in RPMI-1640 media with no FBS at a density of 1x10^5^ cells/200µL/well onto an 8.0 µm pore PET membrane insert (Corning Inc, NY). The insert was placed into a 24 well plate containing 500µL of RPMI-1640 media with 10% FBS supplemented with either 1µM WNT974 or DMSO 1:1000. Cells were allowed to migrate through the membrane overnight, the remaining cells in the top chamber were removed with a cotton tipped applicator, and membranes were then fixed in 4% paraformaldehyde. The fixed cells on the bottom chamber were stained in Richard-Allan Hematoxylin2 (Thermo Scientific) for 3 minutes, then counted.

### Phalloidin assays

Cell lines TC-71, A4573, and SK-ES-1 were plated in RPMI-1640 media with 10% FBS in a density of 2x10^4^ - 2x10^5^ cells/mL/well into chambers of a Lab-Tek II chamber slide system (NUNC, Rochester, NY) and were treated with 1 μM WNT974 or DMSO 1:1000 for 48 hours. Slides were fixed and stained with Cytopainter F-Actin Staining Kit Green (Abcam, Cambridge, UK) per manufacturer’s instructions, and long cytoplasmic extension changes were quantified as previously described [13]. Slides were visualized using an Olympus IX81 motorized inverted microscope and photographed with a Hamamatsu Photonics C9100-02 EMCCD camera using Slidebook software (3i).

### Sarcosphere formation

ES cell lines were cultured in MesenCult (MSC Basal Medium) supplemented with human MSC Stimulatory Supplements (Stem Cell Technologies, Vancouver, BC). The indicated number of cells was seeded on ultra low attachment plates (Corning, Inc, NY) in triplicate wells. Spherical aggregates ≥16 cells were counted in individual wells after 1 week.

### Reverse transcription-polymerase chain reaction (RT-PCR) and qPCR

RNA was extracted from cultured cells or xenografts preserved in RNAlater RNA stabilization reagent (QIAGEN Inc, Valencia, CA) using the RNeasy Mini Kit according to manufacturer’s instructions (QIAGEN Inc, Valencia, CA) and reverse transcribed as previously described (Iscript Reverse Transcriptase, Bio-Rad, Hercules, CA). For quantitative PCR, including PCR arrays, 1 µl cDNA was mixed with 10 µl SSO Advanced Universal SYBR Green SuperMix (Bio-Rad) and 8µl ddH_2_O per reaction with commercially available qPCR primers (Bio-Rad) or preloaded 96 well real-time PCR array plates (Bio-Rad). Primers used include B2M, Axin2, LEF1, TWIST1, SNAI1, ZEB2, and the epithelial to mesenchymal transition SAB target list H96 PCR array, all from Bio-Rad. Quantitative real-time PCR and PCR arrays were performed using a standard two step amplification/melt protocol per manufacturer’s instructions. Quantification of gene expression was performed by the ΔΔC_t_ method, normalized to the housekeeping gene B2M, and quality controls including the Reverse Transcription Control assay (Bio-Rad), DNA Contamination Control assay (Bio-Rad), RNA Quality assay (Bio-Rad) were all performed per manufacturer’s instructions. Gene expression heatmap for PCR arrays were created using the Morpheus software (https://software.broadinstitute.org/morpheus/).

### Lentiviral transduction

Lentiviral production was performed as previously described using the third generation lentiviral packaging system [34]. β-catenin overactivation was achieved by transducing a vector with a S33Y mutant β-catenin cloned into the FUGW backbone as previously described [6].

### Luciferase reporter assay

Cells were seeded in a 24 well plate in triplicates, and were transfected using Lipofectamine 2000 (Invitrogen, Life Technology) with either M50 Super 8x TOPFlash (Addgene plasmid #12456) or M51 Super 8x FOPFlash (Addgene plasmid #12457) reporter construct (kindly provided by Dr. Randal Moon [35]) and pRLSV40 (Promega). After 24 hours of transfection, cells were lysed and analyzed for expression of firefly and Renilla luciferase activity using the Dual-Luciferase Reporter Assay System (Promega) according to manufacturer’s instructions. The TOPFlash/FOPFlash ratio was calculated following normalization by the Renilla luciferase activity from pRLSV40.

### Histopathological analyses

Formalin-fixed, paraffin-embedded tissue was used for analyses. Sections were cut to 5 mm, deparaffinized, and stained with haematoxylin and eosin (Sigma-Aldrich) or processed for immunofluorescence. Samples were deparaffinized in xylene, rehydrated in graded alcohol, and rinsed in PBS, followed by antigen retrieval with boiling citrate buffer, pH 6. Slides were blocked in TBST (1× TBS and 0.05 % Tween 20) with 10 % goat serum. Anti-human mitochondrial antibody (Abcam ab92824, 1:800 dilution) was diluted in TBST and 1% BSA, and slides were incubated overnight in a humidified chamber at 4°C. Slides were washed in TBST prior to application of secondary antibodies of Alexa Fluor 488-conjugated anti-mouse antibodies (Cell Signaling #4408, 1:1000 dilution) in the dark for 1 h at room temperature in a humidified chamber. Slides were then washed in TBST and the coverslips mounted with ProLong Gold antifade reagent with DAPI (Life Technologies) for nuclear counterstaining. Haematoxylin and eosin stained slides were imaged using a Nikon E600 microscope and photographed with a Nikon CCD digital camera using Elements AR software (Nikon), and immunofluorescence was visualized using a Olympus IX81 motorized inverted microscope and photographed with a Hamamatsu Photonics C9100-02 EMCCD camera using Slidebook software (3i).

### Statistical analysis

The statistical significance of the differences in sphere formation and migration was tested using a two-sided Student’s t test. Post-amputation survival and metastasis-free survival of animals were tested for significance by the log-rank test. All statistical analyses were performed using Prism 6 software (GraphPad Software, Inc., La Jolla, CA).

## Abbreviations

ES: Ewing sarcoma
Porcn: Porcupine
EMT: Epithelial-to-mesenchymal transition
NSG: NOD/SCID/IL-2Rγ
PDX: Patient-derived xenograft
CTC: Circulating tumor cell
Fzd: Frizzled
Wnt: Wingless-type MMTV integration site family member

## References

[1] Falk S, Alpert M (1967). Five-year survival of patients with Ewing’s sarcoma. Surg Gynecol Obstet.

[2] Phillips RF, Higinbotham NL (1967). The curability of Ewing’s endothelioma of bone in children. J Pediatr.

[3] Womer RB, West DC, Krailo MD, Dickman PS, Pawel BR, Grier HE, Marcus K, Sailer S, Healey JH, Dormans JP, Weiss AR (2012). Randomized controlled trial of interval-compressed chemotherapy for the treatment of localized Ewing sarcoma: a report from the Children’s Oncology Group. J Clin Oncol.

[4] Ladenstein R, Pötschger U, Le Deley MC, Whelan J, Paulussen M, Oberlin O, van den Berg H, Dirksen U, Hjorth L, Michon J, Lewis I, Craft A, Jürgens H (2010). Primary disseminated multifocal Ewing sarcoma: results of the Euro-EWING 99 trial. J Clin Oncol.

[5] Bienz M, Clevers H (2000). Linking colorectal cancer to Wnt signaling. Cell.

[6] Kahlert UD, Maciaczyk D, Doostkam S, Orr BA, Simons B, Bogiel T, Reithmeier T, Prinz M, Schubert J, Niedermann G, Brabletz T, Eberhart CG, Nikkhah G (2012). Activation of canonical WNT/beta-catenin signaling enhances in vitro motility of glioblastoma cells by activation of ZEB1 and other activators of epithelial-to-mesenchymal transition. Cancer Lett.

[7] Reya T, Duncan AW, Ailles L, Domen J, Scherer DC, Willert K, Hintz L, Nusse R, Weissman IL (2003). A role for Wnt signalling in self-renewal of haematopoietic stem cells. Nature.

[8] Wend P, Holland JD, Ziebold U, Birchmeier W (2010). Wnt signaling in stem and cancer stem cells. Semin Cell Dev Biol.

[9] Liu J, Pan S, Hsieh MH, Ng N, Sun F, Wang T, Kasibhatla S, Schuller AG, Li AG, Cheng D, Li J, Tompkins C, Pferdekamper A (2013). Targeting Wnt-driven cancer through the inhibition of Porcupine by LGK974. Proc Natl Acad Sci U S A.

[10] Navarro D, Agra N, Pestana A, Alonso J, Gonzalez-Sancho JM (2010). The EWS/FLI1 oncogenic protein inhibits expression of the Wnt inhibitor DICKKOPF-1 gene and antagonizes beta-catenin/TCF-mediated transcription. Carcinogenesis.

[11] Scannell CA, Pedersen EA, Mosher JT, Krook MA, Nicholls LA, Wilky BA, Loeb DM, Lawlor ER (2013). LGR5 is Expressed by Ewing Sarcoma and Potentiates Wnt/β-Catenin Signaling. Front Oncol.

[12] Vijayakumar S, Liu G, Rus IA, Yao S, Chen Y, Akiri G, Grumolato L, Aaronson SA (2011). High-frequency canonical Wnt activation in multiple sarcoma subtypes drives proliferation through a TCF/beta-catenin target gene, CDC25A. Cancer Cell.

[13] Endo Y, Beauchamp E, Woods D, Taylor WG, Toretsky JA, Uren A, Rubin JS (2008). Wnt-3a and Dickkopf-1 stimulate neurite outgrowth in Ewing tumor cells via a Frizzled3- and c-Jun N-terminal kinase-dependent mechanism. Mol Cell Biol.

[14] Jin YR, Yoon JK (2012). The R-spondin family of proteins: emerging regulators of WNT signaling. Int J Biochem Cell Biol.

[15] Uren A, Wolf V, Sun YF, Azari A, Rubin JS, Toretsky JA (2004). Wnt/Frizzled signaling in Ewing sarcoma. Pediatr Blood Cancer.

[16] Dieudonne FX, Marion A, Hay E, Marie PJ, Modrowski D (2010). High Wnt signaling represses the proapoptotic proteoglycan syndecan-2 in osteosarcoma cells. Cancer Res.

[17] Pedersen EA, Menon R, Bailey KM, Thomas DG, Van Noord RA, Tran J, Wang H, Qu PP, Hoering A, Fearon ER, Chugh R, Lawlor ER (2016). Activation of Wnt/beta-Catenin in Ewing Sarcoma Cells Antagonizes EWS/ETS Function and Promotes Phenotypic Transition to More Metastatic Cell States. Cancer Res.

[18] Jung HY, Yang J (2015). Unraveling the TWIST between EMT and cancer stemness. Cell Stem Cell.

[19] Ghahhari NM, Babashah S (2015). Interplay between microRNAs and WNT/beta-catenin signalling pathway regulates epithelial-mesenchymal transition in cancer. Eur J Cancer.

[20] Wiles ET, Bell R, Thomas D, Beckerle M, Lessnick SL (2013). ZEB2 Represses the Epithelial Phenotype and Facilitates Metastasis in Ewing Sarcoma. Genes Cancer.

[21] Zhao S, Kurenbekova L, Gao Y, Roos A, Creighton CJ, Rao P, Hicks J, Man TK, Lau C, Brown AM, Jones SN, Lazar AJ, Ingram D (2015). NKD2, a negative regulator of Wnt signaling, suppresses tumor growth and metastasis in osteosarcoma. Oncogene.

[22] Goldstein SD, Hayashi M, Albert CM, Jackson KW, Loeb DM (2015). An orthotopic xenograft model with survival hindlimb amputation allows investigation of the effect of tumor microenvironment on sarcoma metastasis. Clin Exp Metastasis.

[23] Bernstein ML, Devidas M, Lafreniere D, Souid AK, Meyers PA, Gebhardt M, Stine K, Nicholas R, Perlman EJ, Dubowy R, Wainer IW, Dickman PS, Link MP (2006). Intensive therapy with growth factor support for patients with Ewing tumor metastatic at diagnosis: Pediatric Oncology Group/Children’s Cancer Group Phase II Study 9457--a report from the Children’s Oncology Group. J Clin Oncol.

[24] Gaspar N, Hawkins DS, Dirksen U, Lewis IJ, Ferrari S, Le Deley MC, Kovar H, Grimer R, Whelan J, Claude L, Delattre O, Paulussen M, Picci P (2015). Ewing Sarcoma: Current Management and Future Approaches Through Collaboration. J Clin Oncol.

[25] Quail DF, Joyce JA (2013). Microenvironmental regulation of tumor progression and metastasis. Nat Med.

[26] Lawlor ER, Sorensen PH (2015). Twenty Years on: What Do We Really Know about Ewing Sarcoma and What Is the Path Forward?. Crit Rev Oncog.

[27] El-Naggar AM, Veinotte CJ, Cheng H, Grunewald TG, Negri GL, Somasekharan SP, Corkery DP, Tirode F, Mathers J, Khan D, Kyle AH, Baker JH, LePard NE (2015). Translational Activation of HIF1alpha by YB-1 Promotes Sarcoma Metastasis. Cancer Cell.

[28] Fadul J, Bell R, Hoffman LM, Beckerle MC, Engel ME, Lessnick SL (2015). EWS/FLI utilizes NKX2-2 to repress mesenchymal features of Ewing sarcoma. Genes Cancer.

[29] Hatano M, Matsumoto Y, Fukushi J, Matsunobu T, Endo M, Okada S, Iura K, Kamura S, Fujiwara T, Iida K, Fujiwara Y, Nabeshima A, Yokoyama N (2015). Cadherin-11 regulates the metastasis of Ewing sarcoma cells to bone. Clin Exp Metastasis.

[30] Chang YW, Su YJ, Hsiao M, Wei KC, Lin WH, Liang CL, Chen SC, Lee JL (2015). Diverse Targets of beta-Catenin during the Epithelial-Mesenchymal Transition Define Cancer Stem Cells and Predict Disease Relapse. Cancer Res.

[31] Liu H, Yin J, Wang H, Jiang G, Deng M, Zhang G, Bu X, Cai S, Du J, He Z (2015). FOXO3a modulates WNT/beta-catenin signaling and suppresses epithelial-to-mesenchymal transition in prostate cancer cells. Cell Signal.

[32] Zhang Y, Du J, Zheng J, Liu J, Xu R, Shen T, Zhu Y, Chang J, Wang H, Zhang Z, Meng F, Wang Y, Chen Y (2015). EGF-reduced Wnt5a transcription induces epithelial-mesenchymal transition via Arf6-ERK signaling in gastric cancer cells. Oncotarget.

[33] Janku F, Connolly R, LoRusso P, de Jong M, Vaishampayan U, Rodon J, Argiles G, Myers A, Schmitz SF, Ji Y, McLaughlin M, Palmer MR, Morawiak J

[34] Dull T, Zufferey R, Kelly M, Mandel RJ, Nguyen M, Trono D, Naldini L (1998). A third-generation lentivirus vector with a conditional packaging system. J Virol.

[35] Veeman MT, Slusarski DC, Kaykas A, Louie SH, Moon RT (2003). Zebrafish prickle, a modulator of noncanonical Wnt/Fz signaling, regulates gastrulation movements. Curr Biol.

